# Mutation S110L of H1N1 Influenza Virus Hemagglutinin: A Potent Determinant of Attenuation in the Mouse Model

**DOI:** 10.3389/fimmu.2019.00132

**Published:** 2019-02-06

**Authors:** Amelia Nieto, Jasmina Vasilijevic, Nuno Brito Santos, Noelia Zamarreño, Pablo López, Maria Joao Amorim, Ana Falcon

**Affiliations:** ^1^National Center for Biotechnology (CNB-CSIC), Madrid, Spain; ^2^Center for Biomedical Research (CIBER), Madrid, Spain; ^3^Cell Biology of Viral Infection Lab, Instituto Gulbenkian de Ciência, Oeiras, Portugal

**Keywords:** influenza virus, HA S110L mutation, attenuation, *in vivo* pathogenicity, viral entry

## Abstract

Characterization of a pandemic 2009 H1N1 influenza virus isolated from a fatal case patient (F-IAV), showed the presence of three different mutations; potential determinants of its high pathogenicity that were located in the polymerase subunits (PB2 A221T and PA D529N) and the hemagglutinin (HA S110L). Recombinant viruses containing individually or in combination the polymerase mutations in the backbone of A/California/04/09 (CAL) showed that PA D529N was clearly involved in the increased pathogenicity of the F-IAV virus. Here, we have evaluated the contribution of HA S110L to F-IAV pathogenicity, through introduction of this point mutation in CAL recombinant virus (HA mut). The HA S110L protein has similar pH stability, comparable mobility, and entry properties both in human and mouse cultured cells that wild type HA. The change HA S110L leads to a non-significant trend to reduce the replication capacity of influenza virus in tissue culture, and HA mut is better neutralized than CAL virus by monoclonal and polyclonal antibodies against HA from CAL strain. In addition, recombinant viruses containing HA S110L alone or in combination with polymerase mutations considerably increased the LD50 in infected mice. Characterization of the lungs of HA mut infected animals showed reduced lung damage and inflammation compared with CAL infected mice. Accordingly, lower virus replication, decreased presence in bronchioli and parenchyma and lower leukocytes and epithelial infected cells were found in the lungs of HA mut-infected animals. Our results indicate that, mutation HA S110L constitutes a determinant of attenuation and suggest that its interaction with components of the respiratory tract mucus and lectins, that play an important role on influenza virus outcome, may constitute a physical barrier impeding the infection of the target cells, thus compromising the infection outcome.

## Introduction

In 2009 a new influenza A virus from H1N1 subtype, possessing high transmissibility emerged and caused the first pandemic of the twenty-first century (1, 2). The new virus was a reassortant virus containing segments from avian, human, and swine origin and particularly the hemagglutinin (HA) gene of the pandemic virus was closely related to that of the classical swine lineage (3). The HA protein has a pivotal role on influenza virus biology. The receptor binding specificity of the HA is one of the major determinants of viral host tropism, pathogenicity and transmission. The HA is synthesized as a precursor which forms a non-covalent associated homotrimeric precursor (HA0) upon removal of the signal peptide. To activate the membrane fusion and the infectivity of the virus, a proteolytic cleavage of each HA0 monomer in the disulfide-linked subunits, HA1 and HA2, is required (4, 5).

Once internalized influenza virus traffics through the endosomal network, where the endocytosed material is exposed to pH changes. These changes trigger the fusion of HA1/HA2 to undergo irreversible conformational changes that cause membrane fusion with target membranes [reviewed in (6)].

The influenza AH1N1 2009 pandemic virus (AH1N1pdm09) caused mild disease in general, although a substantial number of apparently healthy individuals suffered severe infection, which proposed the coexistence of influenza strains with increased virulence among circulating viruses. Testing this hypothesis, we characterized a pandemic virus isolated from a fatal case patient (F-IAV) that showed increased pathogenicity both, *in vitro* and *in vivo* compared with a virus isolated from a patient with mild symptoms (M-IAV). The F-IAV bears three amino-acids changes that could be responsible for its increased pathogenicity (7). Two of them in the viral polymerase subunits (PB2 A221T and PA D529N) and one in the HA protein (S127L or S110L considering HA1 sequence with or without the signal peptide, respectively). Reverse genetic experiments were performed by introducing the individual changes found in the F-IAV virus, or combination of them in the backbone of the A/California/04/09 to analyze the contribution of each mutation to the overall F-IAV phenotype. Extensive characterization regarding the contribution of polymerase mutations to the exacerbated pathogenicity of the F-IAV virus indicated that change PA D529N was the major contributor for its increased pathogenicity (8). Now we have evaluated the possible contribution of the HA mutation to the increased pathogenicity of the F-IAV virus. Our results indicate that this mutation does not alter major HA recognition by specific antibodies, pH stability, replication in human epithelial cells, or ability for virus entry in human and mouse cell cultures. However, it confers *in vivo* an important attenuation to recombinant viruses carrying individually this change and even in combination with the other pathogenic mutations found in the viral polymerase subunits of the F-IAV. These data indicate that combination of highly pathogenic and attenuation mutations contributed to the final phenotype of F-IAV human isolate (7, 8).

## Materials and Methods

### Ethics Statement

All procedures that required the use of animals performed in Spain complied with Spanish and European legislation concerning vivisection and the use of genetically modified organisms, and the protocols were approved by the National Center for Biotechnology Ethics Committees on Animal Experimentation and the Consejo Superior de Investigaciones Científicas (CSIC) Bioethics Subcommittee (permit 11014). The guidelines included in the current Spanish legislation on protection for animals used in research and other scientific aims (RD 53/2013) and the current European Union Directive 2014/11/EU on protection for animals used in experimentation and other scientific aims were followed. For the experiments that required the use of animals performed in Portugal, all experimental procedures were approved by the Instituto Gulbenkian de Ciência Ethics Committee and the Animal Welfare Body as well as by the Portuguese Authority for Animal Health, Direção Geral de Alimentação e Veterinária (DGAV).

### Biosafety

Cell culture and mouse model experiments performed with recombinant viruses bearing mutations detected in a fatal case IAV were performed in BSL2+ conditions and in a biological insulator in BSL2+ animal facilities, respectively.

### Biological Materials

Cell lines used in this study were MDCK (canine kidney; ATCC CCL-34), NIH 3T3 (mouse embryo fibroblast) and human lung epithelium A549 cells (9). Antibodies rabbit anti-GAPDH, (Sigma), rabbit anti-PB1 (10), rabbit anti-NP (11), and mouse anti-HA (12) were used for western blot assays. Mouse anti-HA and rabbit anti-HA (12) were used for neutralization assay.

### Localization of Mutation in 3D HA Structure

UCSF Chimera 1.10.2 program was used for structural localization of specific mutation in the influenza virus hemagglutinin. Structure of hemagglutinin under accession 3AL4 in Protein Data Bank (PDB) has been used as template.

### Generation of Recombinant HA Mut Viruses

Specific mutations were engineered in pHH plasmids derived from the A/H1N1/California/04/2009 strain using the QuickChange site-directed mutagenesis kit (Stratagene) as recommended by the manufacturer. These materials were developed using the licensed technology (Ref. Kawaoka-P99264US Recombinant Influenza viruses for vaccines and gene therapy). The mutations were rescued into infectious virus by standard techniques as described (13). The identity of rescued mutant viruses was ascertained by sequencing of DNAs derived from the HA, PB2, and PA segments by reverse transcription-PCR (RT-PCR) amplification.

### Neutralization Assays

Blocking infectivity of recombinant viruses by different HA antibodies was performed by Neutralization test. Briefly, viral titer plaque assay was performed on MDCK cells using six different viral multiplicities of infection ranging from 10 to 10^−5^ in the absence or presence of serial dilutions of hybridoma supernatant or serum antibody. Dilutions 1:10; 1:50, 1:100; 1:500, 1:1,000; 1:2,500; 1:5,000; 1:7,500; 1:10,000; 1:25,000, and 1:50,000 were used for monoclonal anti HA antibody. Dilutions 1:500; 1:1,000; 1:2,500; 1:5,000; 1:7,500; 1:10,000; 1:25,000, and 1:50,000 were used for rabbit anti HA serum.

### Western Blotting

For the detection of viral and cellular proteins, total cell extracts were separated by sodium dodecyl sulfate (SDS)-polyacrylamide gel electrophoresis and transferred to Immobilon filters that were saturated with 3% BSA in PBS for 1 h at room temperature. The filters were incubated with primary antibodies diluted in 1% BSA in PBS for 1 h at room temperature (polyclonal antibodies) or overnight at 4° (monoclonal antibodies). After being washed four times for 15 min each with PBS containing 0.25% Tween 20, the filters were incubated with a 1:10,000 dilution of goat anti-rabbit or anti-mouse immunoglobulin G conjugated to horseradish peroxidase. Finally, the filters were washed four times for 15 min each as described above and developed by enhanced chemiluminescence. Dilution 1:1,000 was used for incubation with primary anti PB1, anti-NP, and anti-GAPDH antibodies. Dilution 1:50 was used for monoclonal anti-HA antibody.

### Acid Stability

The acid stability of the viruses was measured by determining viral infectivity after acid treatment. Viruses were diluted in PBS at different pH. The pH was lowered by addition of 0.1 M hydrochloric acid until the desired pH. After incubation at 37°C for 15 min, the viruses were used for titration in MDCK cells by plaque assay.

### Viral Entry

A549 or NIH 3T3 cells were MOCK-infected or infected with CAL or HA mut viruses at 3 pfu/cell. To determine viral entry, A549 or NIH 3T3 cells were treated with 75 or 25 μM chloroquine, respectively, that was added at the same time that the virus inoculum (*t* = 0) or at the indicated times after the start of the infection, with CAL or HA mut viruses. Untreated infected cells were used as reference of infection. The chloroquine treatment was 2 h for all different time points of addition indicated in the corresponding figures (**Figures 3, 4**). All samples were harvested at 8 h post-infection (hpi) and used for NP and GAPDH detection by Western blot. Percentage of NP accumulation in treated cells, compared with untreated cells after normalization by GAPDH levels was determined. Control A549 or NIH 3T3 cells were MOCK-infected or infected with CAL virus for 8 hpi at 3 pfu/cell. These cells were not treated with chloroquine, but suffered the same handling as treated cells to corroborate that the time of addition process does not alter the infection. The experiment was repeated three times with three different viral stocks.

### Viral Growth Kinetics in Cell Culture

Cultured human lung alveolar epithelial cells (A549) were infected at 10^−3^ pfu/cell. Cell supernatants were collected at various times (hours post-infection; hpi), and used for virus titration by plaque assay on MDCK cells.

### RNA Isolation and Q-PCR

RNA isolation from cell cultures was performed with TRIzol [Invitrogen, 15596018)/chloroform (MERCK)] extraction according to manufacturer's instructions. DNA was removed by treatment with DNAse I recombinant, RNase-free (Roche), following instructions of the manufacturer. Quantity and quality analysis of RNA samples were performed by absorbance measuring at 260 nm by *NanoDrop ND*-1,000. All RNA samples were stored at −80°C.

Reverse transcription of RNA samples was performed by *High Capacity RNA Transcriptase Kit* (*Applied System, Thermo Fisher Scientific*) using random primers provided within the kit and following the manufacturer recommendations.

Mx and ISG56 mRNAs were quantify using quantitative PCR approach by imploding *Power SYBR green PCR master mix* (*Applied Biosystems, 4369679*) following the manufacturer recommendations. We used 10% of reverse transcribed cDNA, and 5% of 10 mM specific primers. For estimation of gene expression, we used Thermocycler 7500 Real Time PCR Biosystems 2 min at 50°, 10 min at 94° 40 cycling stages of 15 s at 94° and 1 min at 60°. As a standard curve, plasmids with integrated fragments of ISG56 and MxA genes were used. H 28 SrRNA and m β-actin were used as internal control in each sample.

### *In vivo* Virus Infections

To evaluate pathogenicity of the viruses, five female BALB/c AnNHsd mice (6–7 weeks old) were infected intranasally with different amounts of each recombinant influenza viruses, or were mock-infected. The animals were monitored daily for body weights and survival for 2 weeks. For ethical reasons, mice were euthanized when they presented 25% body weight loss.

For the kinetics experiment, five female BALB/c mice (6–7 weeks old) were infected intranasally with a sublethal dose (10^3^ pfu/50 μl DMEM) of recombinant CAL, or HA mut influenza viruses, or were mock-infected (50 μl DMEM). Mice were euthanized at indicated days post-infection (dpi) by CO2 inhalation and necropsied.

### Viral Titer Estimation in Extracted Organs

Lung samples were homogenized in PBS-0.3%-BSA-penicillin/ streptomycin (100 IU/ml) using an Electronic Douncer (IKA T10 basic, Workcenter). Lung samples were homogenized 1 min at max speed at 4°C and debris were pelleted by centrifugation (2,000 *g*, 5 min, 4°C). Viral titer was determined by standard plaque assay on MDCK cells.

### Histology

Left lung lobes were collected and fixed in 10% buffered formalin for 24 h, then embedded in paraffin, divided into longitudinal sections (3 μm thick) and stained with hematoxylin and eosin. To score lung inflammation and damage, lung samples were screened for the following parameters: interstitial (alveolar septa) inflammation, alveolar inflammation, perivascular/peribronchial inflammation, bronchial exudates, bronchial epithelium hyperplasia, and edema. Each parameter was graded on a scale of 0–4 being 0, absent; 1, very mild; 2, mild; 3, moderate; and 4, severe. The total lung inflammation score was expressed as the sum of the scores for each parameter. Histological scoring was performed blindly by a pathologist.

### Immunohistochemistry

The previously described formalin-fixed, paraffin-embedded tissue specimens were divided into tissue sections (3 μm thick) and processed for immunohistochemical analysis. After deparaffinization of tissue, slides were incubated in sodium citrate buffer, pH 6 at 95°C for 20 min. Sections were permeabilized with PBS supplemented with 0.1% triton X-100 for 7 min, at room temperature and blocked with 2.5% BSA and Fc-block (purified rat anti-mouse CD16/CD32, IGC antibody facility, clone 2.4G2). Infected cells were discriminated by the presence of the viral nucleoprotein (NP) with rabbit α-NP (11) diluted 1:1,000 for 16 h, at 4°C. Endogenous peroxidases were quenched by treating tissue sections with 3% hydrogen peroxide for 15 min, at room temperature. NP positive cells were detected with ImmPRESS HRP Anti-Rabbit IgG (Vector, MP-7401-15), followed by color developing with diaminobenzidine substrate (Roche, 11718096001), both according to manufacturer's instructions. Sections were counterstained with Mayer Hematoxylin before analysis. Tissue sections were observed using a Leica DMLB2 microscope (Leica) and images were captured using NanoZoomer-SQ Digital slide scanner (Hamamatsu). NP expression around bronchioli was scored as: 1, 0–25% infected cells; 2, 25–50% infected cells; 3, 50–75% infected cells; 4, 75–100% infected cells. NP expression was also scored as present/absent infection foci on alveoli. Histological scoring was performed blindly by a pathologist.

### Flow Cytometry

After lung collection, tissue was dissociated by finely chopping using a scalpel blade and digested in a 0.5% (w/v) collagenase D (Roche, 11088858001) solution supplemented with 50 U/ml DNase I (Zymo Research, E1010), in PBS, for 1 h at 37°C. The digested lung was then passed through a 100 μm cell strainer (Falcon, 352360) and centrifuged (650 g, 5 min, 4°C). The pellet was treated with ACK buffer for 4 min, at room temperature. After this incubation, the lung cell suspension was centrifuged and then resuspended in flow cytometry buffer [PBS supplemented with 2% FBS (Life Technologies, 10500-064)]. Cells were counted in each condition and 10^6^ cells transferred to a V-bottom 96 well plate (Thermo Scientific, 249570). Unspecific staining was minimized with Fc-Blocking (purified rat anti-mouse CD16/CD32, IGC antibody facility, clone 2.4G2) for 15 min, at 4°C. Surface staining was performed with α-EpCAM-BV421 (BioLegend, clone G8.8) and α-CD45.2-PE (IGC antibody facility, clone 104.2) diluted 1:100 for 30 min, at 4°C, in the dark. Cells were fixed with IC fixation buffer (Thermo Fisher, 00-8222-49) according to manufacturer's instructions. Fixed cells were washed with permeabilization buffer (0.1% triton X-100 in PBS) and intracellular viral nucleoprotein detected with α-NP (11) diluted 1:100 in the same buffer, for 30 min, at 4°C in the dark. Secondary staining was performed with an α-rabbit IgG conjugated with Alexa Fluor 647 diluted 1:1,000 in permeabilization buffer for 30 min, at 4°C in the dark. Flow cytometry analysis of cell populations was performed in a Becton Dickinson (BD, Franklin Lakes, NJ, USA) LSR Fortessa X-20 SORP equipped with BD FACSDiva™ 8 and FlowJo (Tree Star Inc., Ashland, OR, USA) software. Populations were gated using fluorescence minus one (FMO) controls.

For neutrophils and macrophages quantitation, a similar protocol as indicated above was performed and antibodies used were PerCP-Cy5.5-conjugated CD45 (clon 30-F11) (BioLegend), PeCy7-conjugated CD11b (clon M1/70) (BioLegend), APC-conjugated CD11c (clon N418) (eBIOSCIENCE), and PE-conjugated Ly6G (clon 1A8) (BDBIOSCIENCE). The samples were fixed by incubation with 4% formaldehyde for 20 min, pelleted by centrifugation (700 rmp, 5 min, 4°C) and washed once with PBS. After centrifugation (700 rmp, 5 min, 4°C), cells were resuspended in 0.4 ml PBS and kept at 4°C O/N in the dark. Flow cytometric analysis was performed on a cytometer LSR II (BD Biosciences). Data were analyzed using *CellQuestPro* software.

### Statistics

Student's *t* test and two-way ANOVA were used as indicated in each experiments and Figures. GraphPad Prism v. 5.00 (www.graphpad.com) was used for analysis.

## Results

### Antigenic Properties of HA Mut Virus

**Figure 1:**
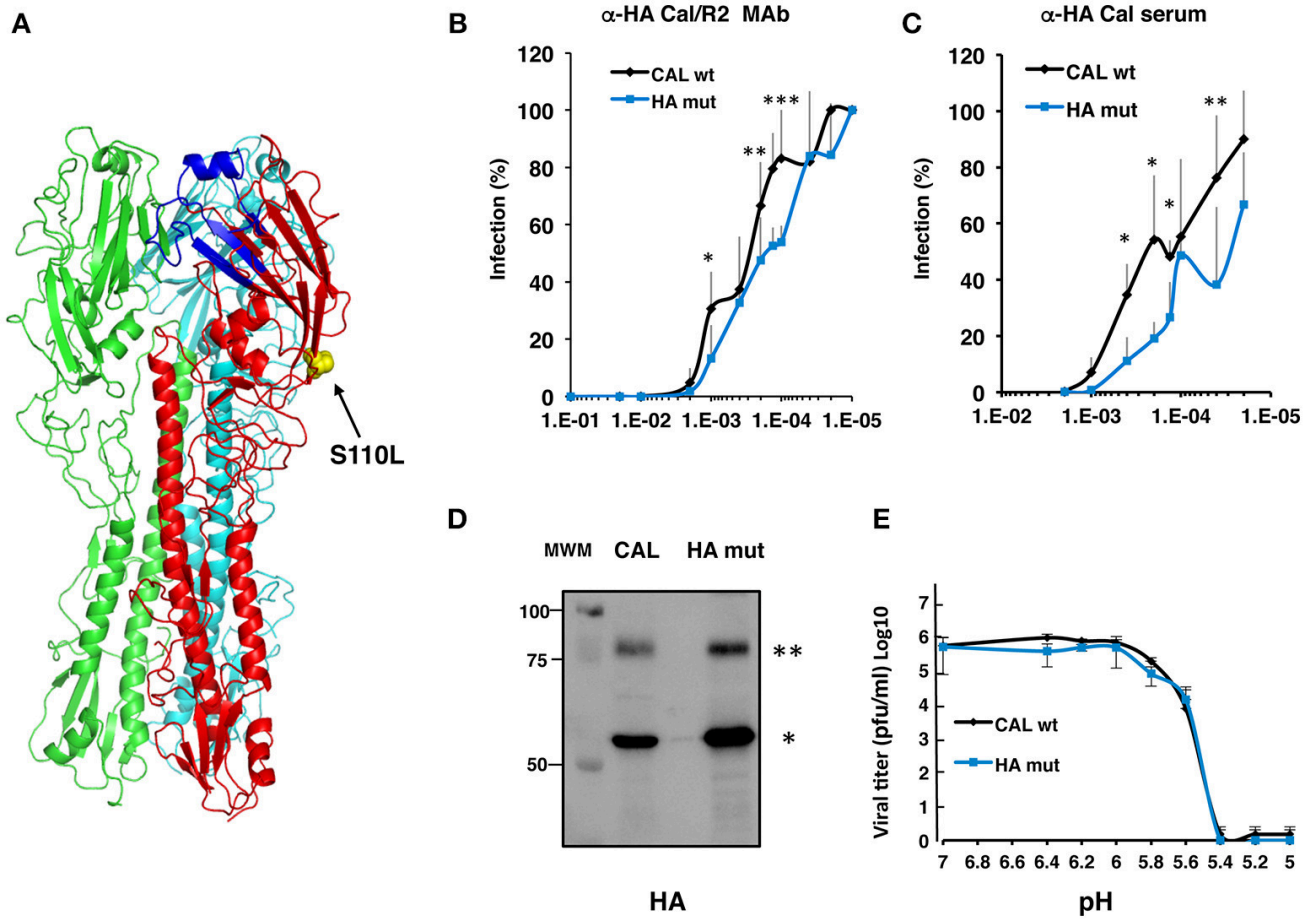
Characterization of HA from CAL and HA mut viruses. **(A)** Structure of HA trimer in Protein Data Bank (PDB) under accession 3AL4 has been used as template for structural localization of HA 110L (yellow) mutation with UCSF Chimera 1.10.2. **(B)** Neutralization assays of MDCK cells infected with Cal and HA mut viruses using serial dilutions of monoclonal antibody α-HA/Cal/2. Significance was determined by two-way ANOVA with Bonferroni *post-hoc* test (^*^p < 0.05; ^**^p < 0.01; ^***^p < 0.001). **(C)** Neutralization assays on MDCK cells infected with CAL and HA mut viruses using serial dilutions of a polyclonal antibody raised against HA from A/California/07/09. The viral infectivity is shown as a percentage of each virus infection in the absence of antibody. The experiments were performed twice and in duplicate using six different viral doses. Significance was determined by two-way ANOVA with Bonferroni *post-hoc* test, ^**^p < 0.01. **(D)** Movility of HA. CAL or HA mut viruses were applied to SDS-gels, followed by Western bot assays using α-HA/Cal/2 monoclonal antibody. (^**^) denotes HA0, and (^*^) HA1. **(E)** pH stability of HA. CAL and HA mut viruses were incubated at the indicated pHs and their replication capacity evaluated in MDCK cells by plaque assay. The experiment was performed in triplicates and each plaque assay was performed twice. Significance was determined by two-way ANOVA with Bonferroni *post-hoc* test, ns (not significant).

First, we localized position HA 110 in the three-dimensional HA structure (14) (Figure 1A). As shown, it represents an exposed residue that is placed at the globular part of the HA protein, in a region that has not been previously reported as involved on HA functional modulation. Characterization of antibody recognition of recombinant virus bearing HA S110L mutation was performed by neutralization assays. Two different specific antibodies that recognize HA protein were used; a monoclonal antibody generated against A/California/07/09 strain that reacts with different 2009 pandemic viruses but does not recognize H1N1 viruses isolated before 2009 (α-HA/Cal/2) (12) and a rabbit polyclonal antibody generated using as antigen a recombinant vaccinia virus expressing the HA protein from A/California/07/09 strain (12). As reported, amino acid changes S88Y and K136N are needed simultaneously to inhibit binding of α-HA/Cal/2 to A/Cal/07/09 HA suggesting that at least two different but overlapping epitopes are recognized by the antibody (Supplementary Figure 1) (12). In addition, change T89K causes a fully resistant protein to neutralization by the antibody (Supplementary Figure 1) (12). MDCK cells were infected with recombinant virus A/California/04/09 (CAL) or the CAL recombinant virus bearing the HA S110L point mutation (henceforward HA mut) at serial multiplicity of infection (moi) ranged between 10^−5^ and 10 in the presence of serial dilutions of each anti-HA antibody. After viral adsorption, infected cells were incubated in the absence or presence of the same antibody dilutions in semi-solid media for 48 h and data were analyzed by plaque quantitation. The results showed that HA mut virus is recognized and neutralized better than CAL virus by the HA monoclonal antibody (Figure 1B). Slightly better recognition was found using the polyclonal antibody (Figure 1C), indicating that the HA S110L mutation may elicit some structural changes in the HA protein.

### HA Post-translational Modifications

The HA protein binds to the receptor on the cell and fuses with cellular membrane; it is known that receptor binding and fusion activation are modulated by HA glycosylation (15). HA glycosylation plays an important role in influenza viruses life cycle (16) and the structural integrity requires particular glycosylation sites. In addition, glycosylation of an antigenic epitope can prevent antibody binding (17). HA can undergo *N*-linked glycosylation through *N*-glycosidic linkages to the Asn residue of the glycosylation motif Asn-X-Ser/Thr-Y, where X/Y may represent any amino acid except proline (18). HA can also be O-glycosylated through the addition of N-acetyl-galactosamine to serine or threonine residues; it has not been reported clear consensus sequence motifs for prediction and identification of O-glycoproteins (19). Amino acid 110 of HA of A/California/04/09 is not located at a putative N-glycosylation motif. However, this amino acid is changed from S to L in HA mut and it could represent an O-glycosylation motif or could modulate N-glycosylation mediated by induced conformational changes. To examine possible differences in HA glycosylation between CAL and HA mut, we analyzed the HA mobility from CAL and HA mut viruses by SDS-gel electrophoresis and Western blotting using α-HA/Cal/2 monoclonal antibody. No appreciable changes in the HA mobility between wild type and mutant HA were detected (Figure 1D), suggesting that no major glycosylation changes occur.

### HA S110L Acid Stability

The HA protein binds to the receptor on the cell and mediates the low pH-triggered viral and cellular membrane fusion. It has been reported that the activation energy of fusion measured as the pH activation of HA (acid stability) is linked to a plethora of viral functions such as pathogenicity, adaptation, host-range, and transmissibility (20–23). To examine possible differences on acid stability between CAL and HA mut that could modulate virus fitness, both viruses were incubated at different pHs as indicated in Materials and Methods and their replication capacity evaluated in MDCK cells by plaque assay. Buffers adjusted to pH range from 5.4 to 6.4 with increments of 0.2 units were used. Both viruses showed the highest replication capacity until treatment with pH 5.8 (Figure 1E). These results indicate a similar acid stability of both viruses and are in agreement with previous reports of pH stability of 2009 pandemic viruses (20).

### Biological Characterization of HA Mut Virus in Cell Culture

#### Binding and Entrance of HA Mut Virus

**Figure 2:**
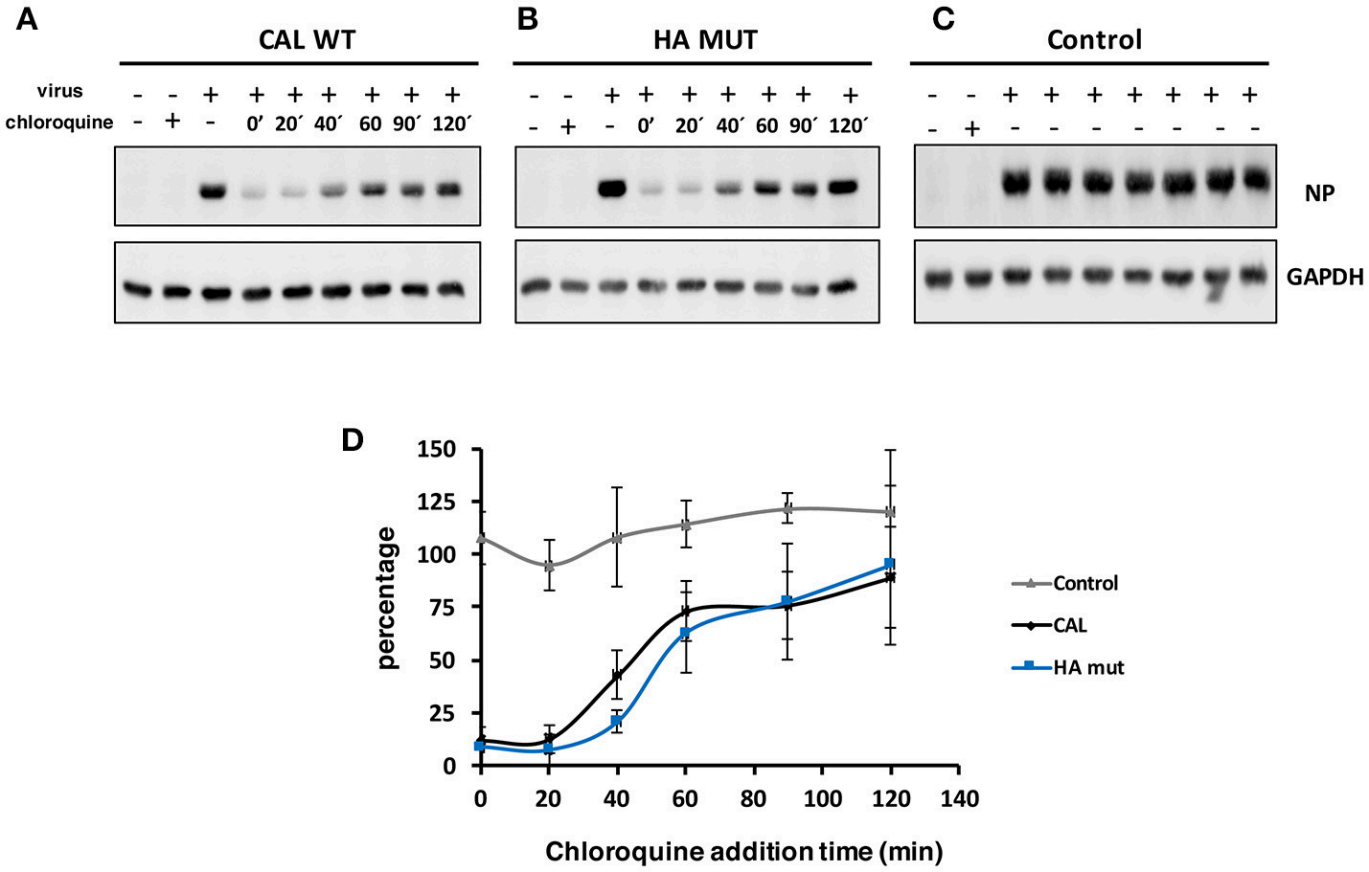
CAL and HA entry in A549 cells. A549 cells were infected with 3 pfu/cell with **(A)** CAL, **(B)** HA mut, or **(C)** CAL control viruses and left untreated (–) or treated with 75 μM chloroquine (+) that was added at the indicated times of the infection and was left for 2 h after each indicated time of chloroquine addition. Then, chloroquine was removed and the infection continued until 8 hpi. Infected cells were harvest at 8 hpi and used for NP and GAPDH detection by Western blot. A representative image of each condition is shown. **(D)** Ratios of NP/GAPDH accumulation in treated cells compared with untreated cells in parts A and B, or compared with 0 hpi in part **(C)** of three independent experiments using different viral stocks are shown. NP/GAPDH ratio obtained in cells untreated and infected was taken as 100%. The experiment was performed in triplicates with two different viral stocks. Significance was determined by two-way ANOVA with Bonferroni *post-hoc* test, (ns) not significant.

**Figure 3:**
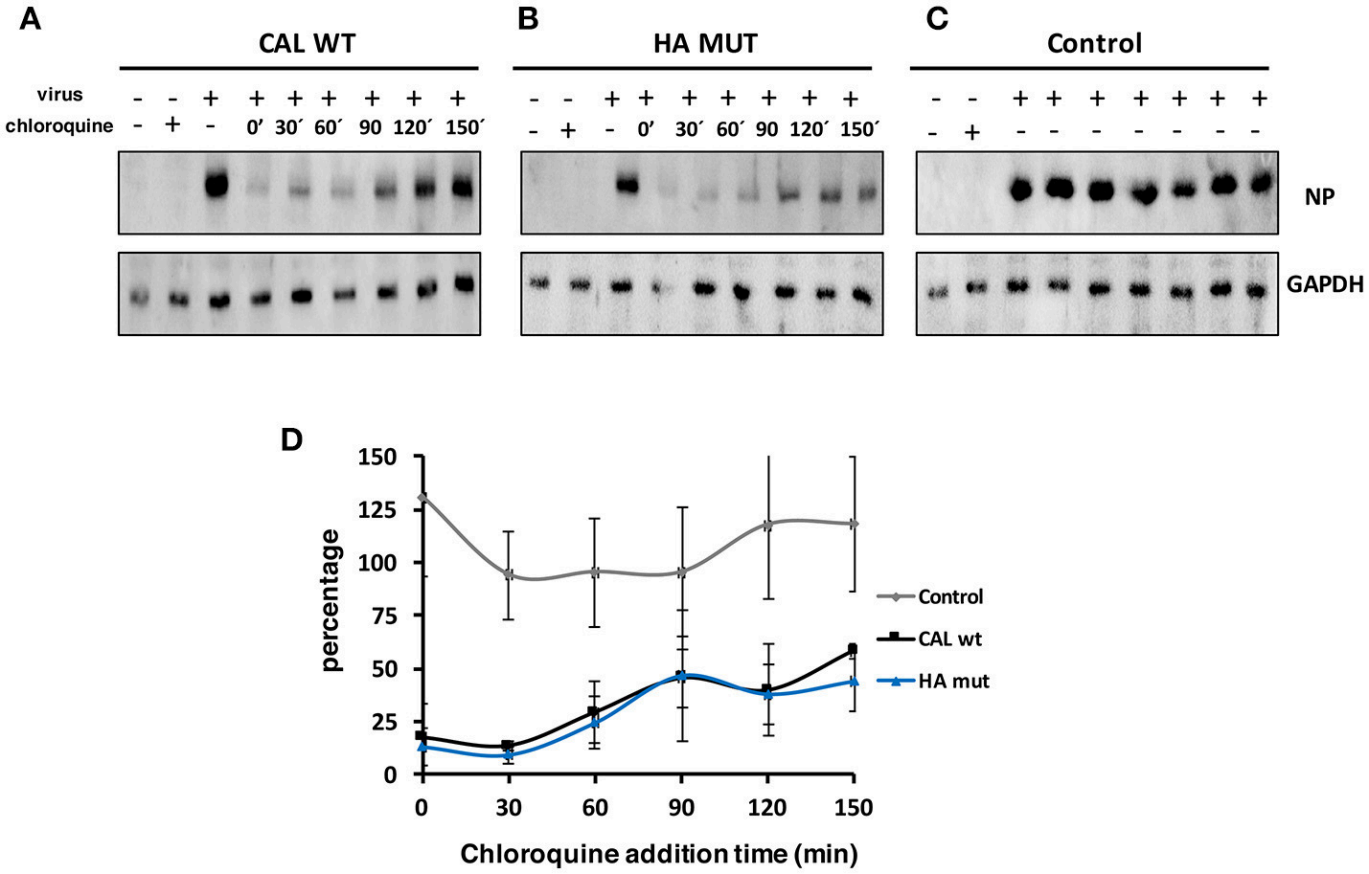
CAL and HA entry in NIH3T3 cells. NIH3T3 cells were infected with 3 pfu/cell with **(A)** CAL, **(B)** HA mut, or **(C)** CAL control viruses and left untreated (–) or treated with 25 μM chloroquine (+) that was added at the indicated times of the infection and was left for 2 h after each indicated time of chloroquine addition. Then, chloroquine was removed and the infection continued until 8 hpi. Infected cells were harvest at 8 hpi and used for NP and GAPDH detection by Western blot. A representative image of each condition is shown. **(D)** Ratios of NP/GAPDH accumulation in treated cells compared with untreated cells in parts **(A,B)**, or compared with 0 hpi in part **(C)**, of three independent experiments using different viral stocks are shown. NP/GAPDH ratio obtained in cells untreated and infected was taken as 100%. The experiment was performed in triplicates with two different viral stocks. Significance was determined by two-way ANOVA with Bonferroni *post-hoc* test, (ns) not significant.

Hemagglutinin plays a major role on viral tropism mainly mediated by receptor binding specificity. To compare the properties of HA mut with CAL virus, on human and mouse cells, cultures of human alveolar lung epithelial cells (A549) and mouse embryonic fibroblasts (NIH 3T3) were used to examine viral entry (Figures 2, 3). Chloroquine is a lysosomotropic base that inhibits endocytosis of influenza virus (24, 25) preventing viral entry. A549 and NIH 3T3 cells were infected with CAL or HA mut viruses at an moi of 3 and cells were left untreated or treated with chloroquine at 75 or 25 μM, respectively, at different times after infection (minutes). Following 2 h of treatment after each indicated time of chloroquine addition, chloroquine was removed. Samples were recovered at 8 hpi for all conditions tested and accumulation of NP and GAPDH determined by Western blot analysis. As control A549 or 3T3 cells were infected with CAL virus and were not treated with chloroquine but they suffer the same handling of media change than treated cells (Figures 2C, 3C). These data showed that the management of the cells during the experiment does not alter viral infection. Images representing protein accumulation and quantitation of the NP/GADPH ratios of three independent experiments performed in A549 cells (Figure 2) or NIH3T3 cells (Figure 3) are shown. Similar curves of viral protein accumulation on CAL- and HA mut-infected A549 cells were observed. Only a slight difference after 40 min of chloroquine addition was observed but with no statistical significance. Regarding NIH3T3 cells, parallel viral accumulation curves were obtained. These results indicate that viral entry and replication of CAL and HA mut are comparable both in human and mouse cells. Therefore, HA S110L change maintains similar viral entry ability to that of control CAL virus.

#### Growth Kinetics of CAL and HA Mut Viruses

**Figure 4:**
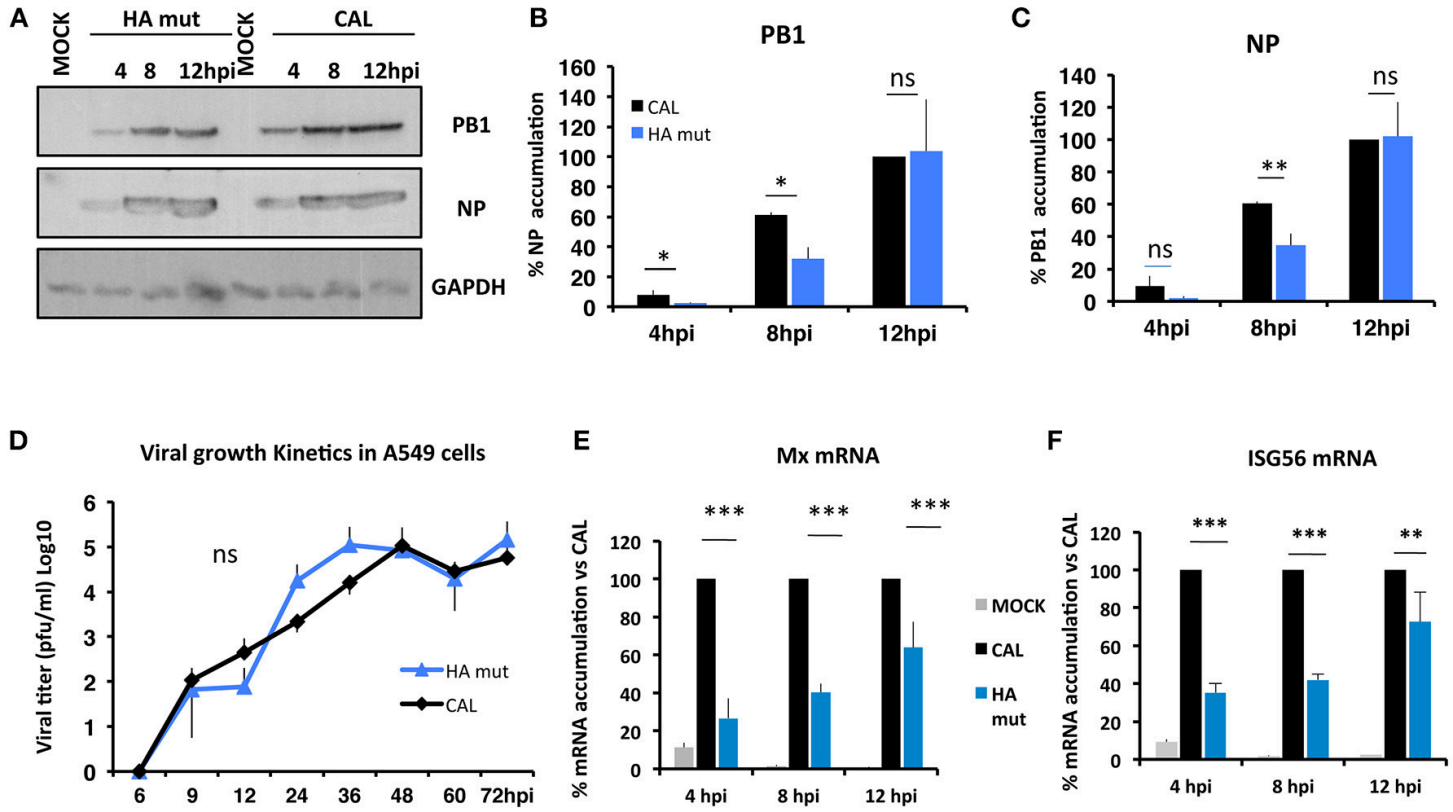
Growth kinetics of CAL and HA mut viruses. **(A)** Cultured human lung epithelial cells (A549) were infected with CAL and HA mut viruses 3 pfu/cell. At the indicated times, aliquots were used for Western blot assays to detect PB1, NP, and GAPDH proteins. The experiment was performed in triplicates and one representative data is shown. **(B,C)** show quantification and significance analysis of triplicates. Data are shown as % related to maximum accumulation of viral proteins in CAL virus infected cells. Values shown as means and error bars indicate mean ± SD of three independent experiments (ns Not significant, ^*^p < 0.05, ^**^p < 0.01 by *t-*Student test). **(D)** A549 cells were infected at 10^−3^ pfu/cell, at the indicated times, aliquots were taken and used for titration on MDCK cells. The experiments was repeated three times and significance was determined by two-way ANOVA with Bonferroni *post-hoc* test, (ns) not significant. **(E)** Cultured human lung epithelial cells (A549) were mock infected or infected with CAL, or HA mut virus stocks at moi 0.5. At indicated hours post-infection (hpi), samples were used to evaluate Mx mRNA **(E)** or ISG56 mRNA **(F)** by q-PCR. Data are shown as % of mRNA accumulation in CAL infected cells at each time tested. Quantification and significance analysis of triplicates are shown as means and error bars indicate ± SD (^**^p < 0.01, ^***^p < 0.001 by *t-*Student test).

Next, we analyzed the replication capacity of these viruses in *in vitro* assays. Cultures of human alveolar lung epithelial cells (A549) were infected with CAL and HA mut viruses at high (3 pfu/cell) (Figure 4A) and low (10^−3^ pfu/cell) (Figure 4D) moi.

Accumulation of viral proteins in a single cycle replication assay was used to determine viral growth kinetics. Total cell extracts were obtained at indicated hours post-infection (hpi) and accumulation of viral proteins was monitored by western blotting (Figures 4A–C). HA mut virus infected cells accumulated lower levels of PB1 polymerase subunit than CAL infected cells at 4 and 8 hpi (*p* < 0.05) (Figures 4A,B). A difference in NP accumulation was observed at 8 hpi (*p* < 0.01) (Figure 4C). However, no differences were found between the wild type and the mutant virus at 12 hpi (Figures 4A–C). In multiple cycles assay, we determined the viral titers at different hours post-infection (hpi); although HA mut showed a slight delay on viral replication at early hpi, this data is not statistically significant and both viruses reached similar titers at later times (Figure 4D). Thus, the change HA S110L leads to a non-significant trend to reduce the replication capacity of influenza virus in tissue culture.

#### Antiviral Response of CAL and HA Mut Viruses

Next, the induction of the antiviral response by CAL and HA mut viruses was evaluated by infection of A549 cells at 0.5 moi, monitoring the accumulation of antiviral Mx and ISG56 mRNA by RT-PCR at 4, 8, and 12 hpi. HA mut infected cells accumulate lower amount of Mx than CAL virus infected cells (3.3-, 2.5-, and 1.5-fold, *p* < 0.001, respectively) (Figure 4E). Similar results were obtained for accumulation of ISG56 in HA mut infected cells compared to CAL infected cells (2.7-, 2.5-, and 1.3-fold, *p* < 0.001, respectively) (Figure 4F). These data indicate an impaired induction of the antiviral response elicited by HA mut virus and suggest a delay in an early step in the infection cycle.

### Contribution of the HA S110L Mutation to *in vivo* Pathogenicity

Since influenza virus polymerase is one of the major virulence determinants of influenza virus, we previously characterized the contribution of the polymerase subunits mutations found in the fatal F-IAV virus to its high pathogenicity. Using recombinant viruses that express individually each of the changes found in the polymerase subunits, PB2 A221T (PB2 mut), PA D529N (PA mut), or both PB2/PA mut, we observed similar replication capacity of these viruses in A549 culture cells although they showed differences in antiviral response activation in infected cells (8). However, mutation PA D529N highly increased virus pathogenicity *in vivo* and the lethal dose 50 (LD50) of PA mut compared with the LD50 of PB2 mut showed a reduction of more than 3 log units (8). These results indicate that virus replication *in vitro* does not precisely represent viral fitness in *in vivo* model; a much more complex situation for virus life cycle.

**Figure 5:**
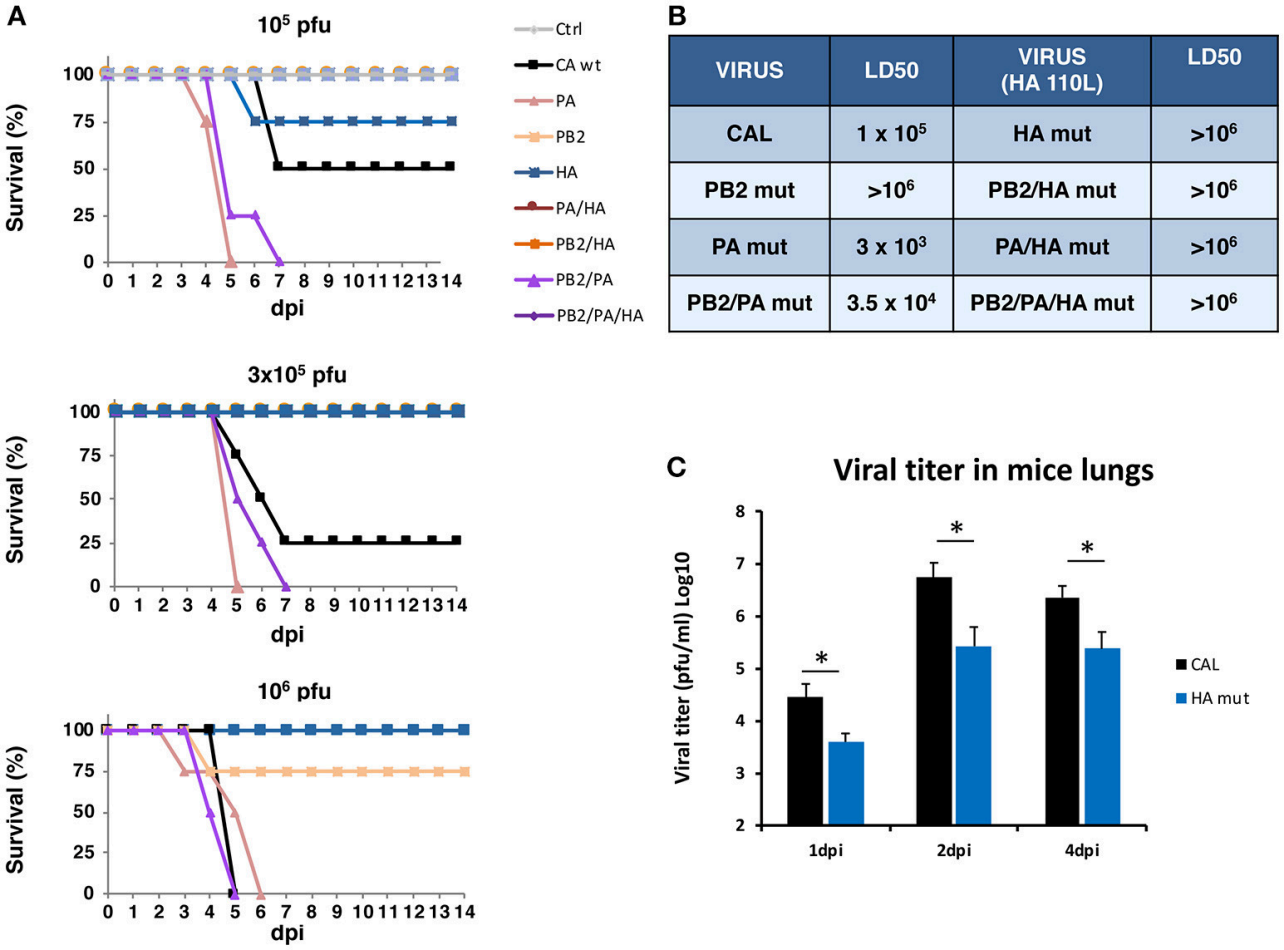
*In vivo* pathogenicity of HA mut virus. Mice (*n* = 5) were inoculated intranasally with 10^6^-10^2^ pfu of each recombinant virus. **(A)** Survival curves. Some series corresponding to attenuated viruses appear overlapped at 100% together with MOCK infection data. **(B)** The dose that caused 50% mortality of mice in each infection (LD50) is shown. **(C)** At indicated dpi, viral titers were determined in the lungs (pfu/g tissue). Significance was determined by Student's *t* test (^*^p < 0.05).

To examine the contribution of HA S110L mutation to the F-IAV pathogenicity, in addition to the HA mut virus, we generated additional recombinant viruses carrying the HA S110L mutation in combination with the mutations found in the polymerase subunits of the F-IAV virus (Table 1) and evaluated the *in vivo* pathogenicity. The recombinant viruses were used to infect mice with various virus doses or with DMEM as control, survival and body weight were monitored daily for 2 weeks and the LD50 for each virus was determined. For better comparison survival (Figure 5A), LD50 (Figure 5B) and body weight (Supplementary Figure 2) data of both previous (8) and current recombinant viruses are shown. A clear increase on survival was found in mice infected with HA mut compared with those infected with CAL virus, as well as in mice infected with any recombinant bearing HA S110L mutation compared with that containing HA 110S. Accordingly, an important increase in the LD50 of all viruses containing HA S110L change was observed. As reported (8), CAL, PB2 mut, PA mut, and PB2/PA mut viruses showed an LD50 of 10^5^, >10^6^, 3 × 10^3^ and 3.5 × 10^4^, respectively, while HA mut and any of the recombinant viruses that bear HA S110L mutation showed an LD50 higher than 10^6^ (Figure 5B compared left and right columns). This fact even occurs in the case of mice infected with a recombinant virus that also contains PA D529N mutation that works as an extremely pathogenicity determinant (Figure 5B compared PA mut and PA/HA mut) indicating that HA S110L change is a very potent determinant of attenuation.

**Table 1:** Sequences of the recombinant viruses at position PA 529, PB2 221, and HA 110.

**Recombinant virus**	**PA 529**	**PB2 221**	**HA 110**
CAL	D	A	S
PB2 mut	D	T	S
PA mut	N	A	S
HA mut	D	A	L
PB2/PA mut	N	T	S
HA/PB2 mut	D	T	L
HA/PA mut	N	A	L
HA/PA/PB2 mut	N	T	L
F-IAV	N	T	L
M-IAV	D	A	S

*Sequences of the different recombinant viruses and M-IAV and F-IAV isolates at the indicated position is shown*.

### Characterization of the Lungs of Mice Infected With CAL and HA Mut Viruses

The above results showed that HA S100L mutation causes a significant reduction on virus pathogenicity; thus we further characterized the possible reasons for this attenuated phenotype. To that aim, mice were infected with CAL or HA mut viruses at sub-lethal dose (10^3^ pfu) or were mock-infected. Samples were recovered at several dpi and viral titers determined in the lungs (Figure 5C). According with the observed LD50, the attenuated HA mut virus showed lower titers in lungs at any dpi than those found in the lungs of CAL- infected mice.

#### Histological Damage

**Figure 6:**
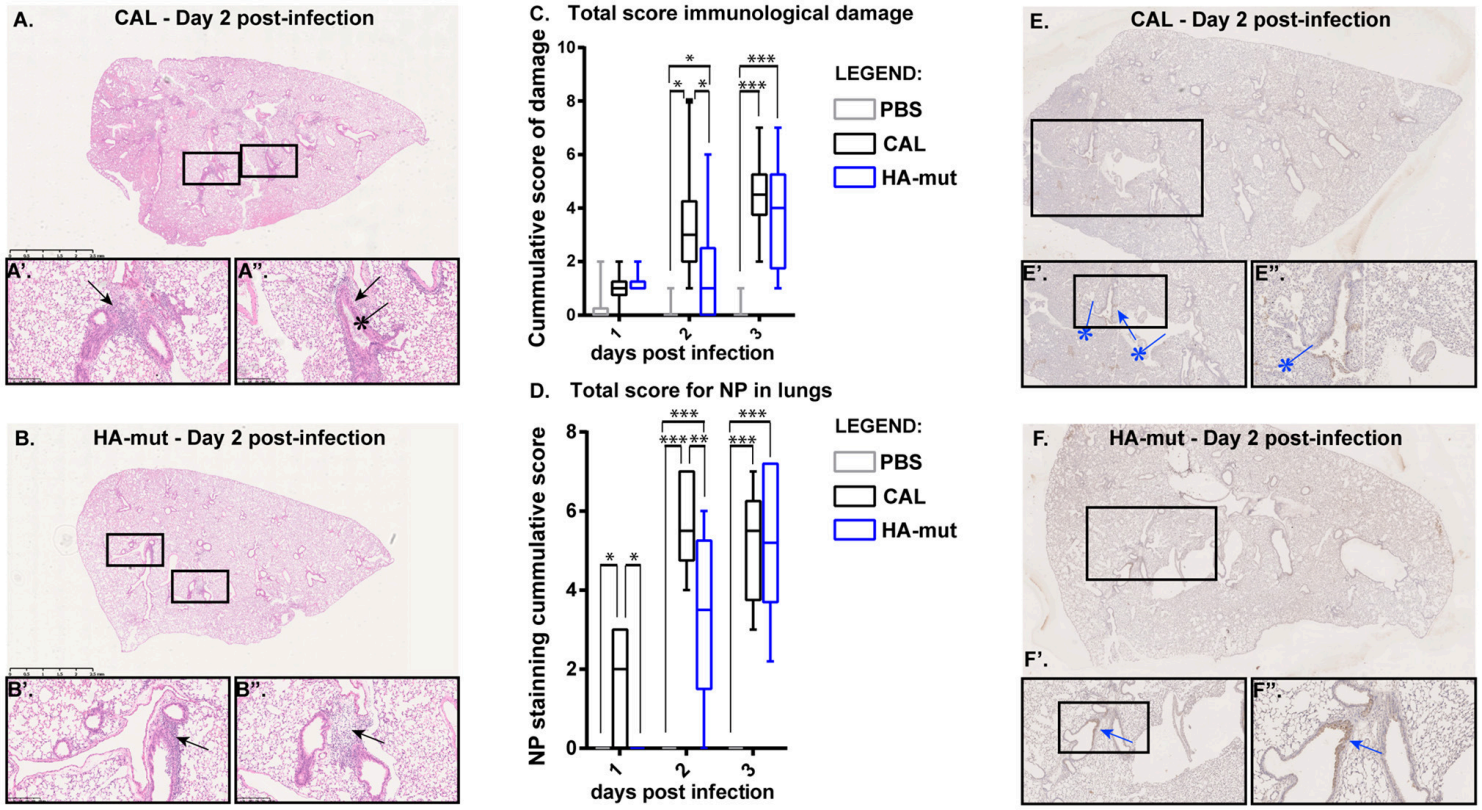
Immunological damage and NP expression in lungs of CAL and HA mut-infected mice. Five Balb/c female mice/condition of 6–9 weeks of age were infected with 10^3^ pfu of CAL and HA-mut viruses or mock infected. At days 1, 2, and 3 lungs were collected, fixed with formalin, processed for histological analyses, and stained with H&E. **(A,B)** Show representaitive lungs at 1.25X amplification for day 2 p.i. where higher differences were detected. Inlets are areas 10× amplified where specific damage (or its absence) is observed. (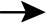 Perivascular/peribronchioli infiltrates; 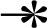 Bronchial exudates). **(C)** Different inflammation and damage parameters were graded on a scale 0–4 (0, absent; 1, very mild; 2, mild; 3, moderate; and 4, severe). The total “lung inflammation score” was expressed as the sum of the scores for each parameter depicted in Supplementary Figure 2 and plotted as box-to-whiskers graph from min to max and line represents the median. **(D–F)** The same lungs were also processed for NP staining. **(E,F)** show representative lungs at day 2 with 1.25X amplification. Inlets are areas 5–20× amplified where staining (or its absence) is observed. (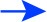 Perivascular/peribronchioli infected areas; [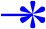] parenchyma areas infected). **(D)** NP expression in lungs was scored for all parameters evaluated depicted in Supplementary Figure 3 and plotted as a box-to-whiskers plots graph min to max with the line representing the median. Statistical analyses was done using two-way ANOVA and is indicated as ^*^p < 0.05; ^**^p < 0.01, ^***^p < 0.001 where significant differences were found. The experiment was performed twice.

Different parameters that contribute to lung damage and inflammation were evaluated in histological preparations of CAL- and HA mut infected lungs at different dpi. Histological examination of lungs of MOCK- CAL- and HA mut- infected mice and damage parameters that include perivascular/peribronchial infiltration, bronchial exudates, edema and interstitial inflammation are shown in Supplementary Figure 3. A representative image of histological preparations of lungs of CAL- and HA mut-infected animals and the total “lung inflammation score” that is expressed as the sum of the scores for each parameter are presented in Figures 6A–C. In agreement with the reduced lung viral titers, a significant decrease on peribronchial infiltration (2 dpi) and bronchial exudates (3 dpi) damage was found in HA mut infected mice (Supplementary Figures 3J,K). In addition, significant but mild damage in bronchial exudates and edema (2 dpi) was observed in CAL- infected mice when compared with MOCK animals, but absent in HA mut infected animals (Supplementary Figures 3K,E)

#### Virus Localization

Next, we performed immunohistochemical analysis to examine the presence of the viruses at different lung structures such as bronchioli and parenchyma (Figures 6D–F). Paraffin-embedded tissue specimens were divided into tissue sections (3 μm thick) and processed for NP detection as described in Materials and Methods. The bronchioli are one of the smallest airways in the respiratory tract, and go directly to the alveolar canals, which contain the alveoli responsible for exchanging gases with the blood. The lung parenchyma comprises a large number of thin-walled alveoli, which serves to maintain proper gas exchange. Images displaying localization of viral NP in bronchioli and parenchyma of infected mice are shown in Supplementary Figures 4A–I. A significant reduction on the number of infected bronchioli was found at 2 dpi in HA mut infected animals compared with CAL infection, as well as significant differences between CAL- and MOCK-infected mice that were absent in HA mut (Supplementary Figure 4J). Despite there were no significant differences observed in the percentage of infection of each bronchioli between CAL- and HA mut-infected mice (Supplementary Figure 4K), significant infected parenchyma was found in CAL-infected mice compared with MOCK animals at 2 dpi and absent in HA mut mice (Supplementary Figure 4L). Representative images of CAL and HA mut localization in bronchioli and parenchyma as well as the total score of infection are shown in Figures 6D–F. A significant reduction was found in HA mut infected animals at 2 dpi compared with wild type virus. In addition, CAL virus was more abundant in lung structures at 1 dpi than HA virus (Figure 6D), indicating that this virus reaches the lungs at higher levels.

### Lung Cells Infected by CAL and HA Mut Viruses

**Figure 7:**
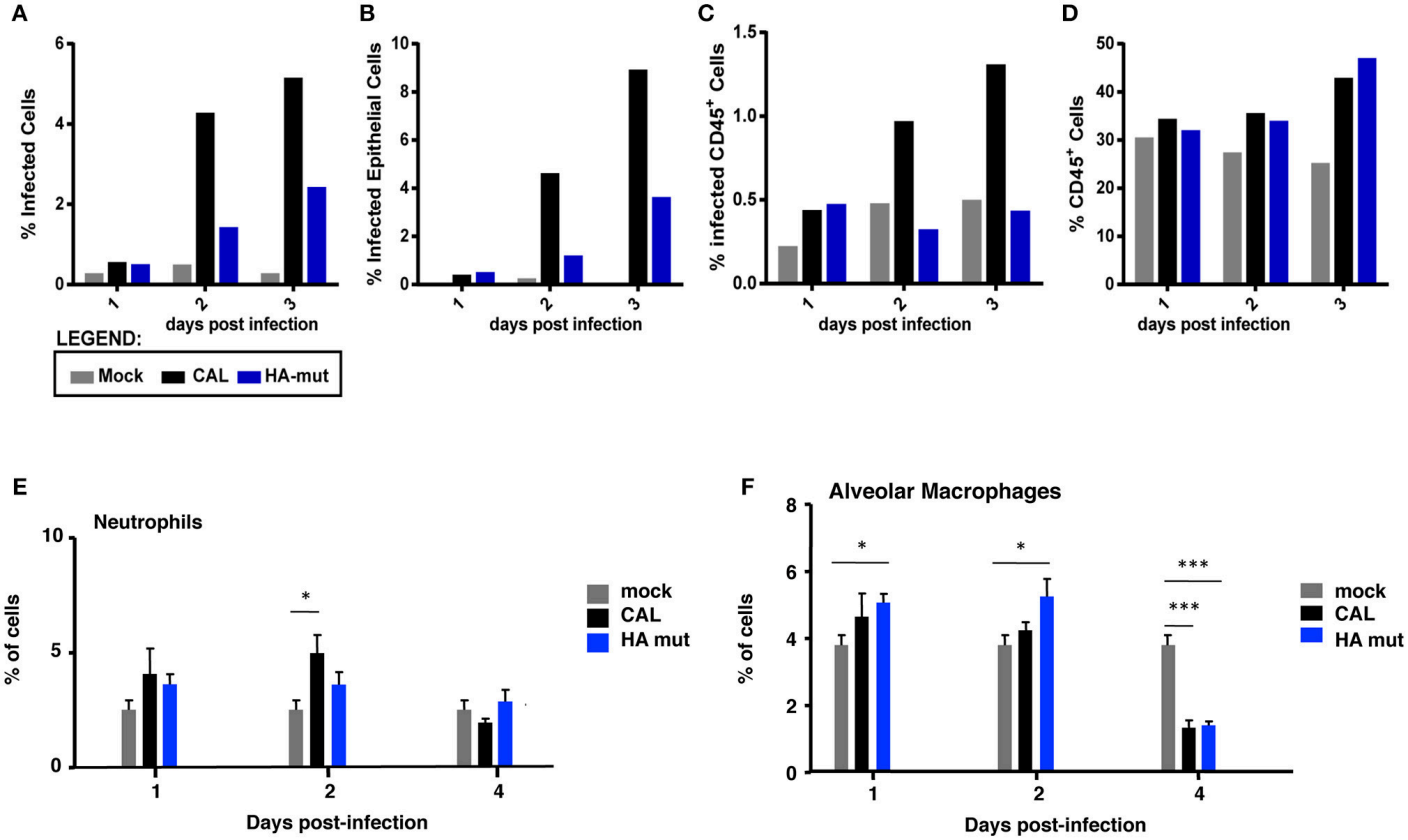
Lung cells infected by CAL- and HA mut viruses. Five Balb/c female mice/condition of 6–9 weeks of age were infected with 10^3^ pfu of CAL and HA-mut viruses or mock infected and pooled into one sample. At days 1, 2, and 3 post-infection lungs were collected, and processed for flow cytometry. Single cell suspension was stained with antibodies against NP, EpCam and CD45, to detect infected cells, epithelial cells and leukocytes, respectively. **(A)** Shows events positive for NP in the y-axis. **(B,C)** Percentage of specific populations of infected cells in relation to the total events, total number of gated EpCam or of CD45 positive cells were plotted. **(D)** Total percentage of CD45 positive cells, regardless of the infectivity state were plotted. The experiment was performed twice and overall 5 mice per condition pooled into one sample. **(E,F)** Mice (*n* = 5) were inoculated intranasally with a sublethal dose (10^3^ pfu) of each recombinant virus or PBS (MOCK) as control and were evaluated in three groups. Neutrophils **(E)** and alveolar macrophages **(F)** were quantified in the lungs (% of cells) at indicated dpi. Significance was determined by Student's *t*-test (^*^p < 0.05, ^***^p < 0.001).

Virus pathogenicity depends on the nature of the virus, the host conditions and the adequate response to the infection. The above results showed higher lung tissue damage induced by the CAL virus, compared to HA mut virus- infected mice. Leukocytes are the cells that detect pathogens in lungs and trigger the immune response against the infection. To examine possible differences on host response, we monitored the infection of alveolar leukocytes as well as epithelial cells in CAL and HA mut- infected lungs and the influx of leukocytes at several days of infection by flow cytometry analysis (Figure 7). For that, 5 Balb/c mice were infected with 10^3^ pfu and at days 1, 2, 3 lungs were collected, and the cell suspension analyzed by flow cytometry analysis using antibodies that recognize infected cells (NP), epithelial cells (EpCam) or leukocytes (CD45). A significant decrease of total infected cells in HA mut infection was observed (Figure 7A), that corresponded to a decreased amount of infected epithelial cells (Figure 7B) and leukocytes (Figure 7C) at every day post-infection. Examination of recruited leukocytes showed similar amounts on CAL and HA mut infected mice (Figure 7D). In addition, we monitored the presence of neutrophils and alveolar macrophages in mock, HA mut, or CAL- infected lungs at several days after sub-lethal infection (Figures 7E,F). HA mut induced lower infiltration of neutrophils and higher presence of alveolar macrophages than CAL virus at 2 dpi in the lungs of infected animals. Although these differences are not statistically significant (Figures 7E,F), they agree with the scenario of having more cells infected, including CD45 positive cells, that are very likely eliminated at day 2 and 3 post-infection (Supplementary Figure 3J). These two features in immune response, lower infiltration of neutrophils and higher presence of macrophages partially observed for HA mut virus compared to CAL, have been described as essential factors for attenuated influenza virus infections (26, 27) and agree with the role of HA S110L change in attenuation *in vivo*.

Together, these results indicate that the attenuated phenotype of the recombinant HA mut virus is the consequence of a lower infection of the target cells in the mice, despite its ability to efficiently replicate and enter in cultured cells.

## Discusion

During the 2009 influenza pandemic, particularly virulent isolates were described, such as one that was isolated from a fatal case young patient (F-IAV), that contained three-point exclusive mutations, two in the viral polymerase subunits (PA D529N and PB2 A221T) and one in the HA protein (S110L) (7). Extensive characterization of these mutations studied individually, showed that PB2 A221T mutation, is a determinant of attenuation (8). In contrast, mutation PA D529N, was characterized as an extremely high pathogenicity determinant (8).

There is considerable interest in identifying and characterizing mechanisms of viral attenuation, which could involve several steps in the replication cycle of the virus and in circumventing the barriers to establish the infection in the infected host. In fact, the most efficient viral vaccines use live attenuated viruses (28, 29). In particular, those affecting the viral protein HA are of special interest, as HA is the major epitope displayed to the immune system and constitutes an important determinant of pathogenicity of influenza viruses. One window of opportunity comes from understanding attenuation at the genetic level, which was done in this work using engineered viruses and enabled us to identify a new determinant of attenuation in HA.

HA protein is responsible for the initiation of the infection, through recognition of cell surface receptors followed by membrane fusion. Several of its properties have a central role controlling the infection, such as the receptor binding and fusion capacity that are modulated by HA glycosylation and are dependent on cleavage activation by host proteases (15). Once internalized, the endocytosed virus is exposed to acidic pH that triggers membrane fusion and allows the dispersion of viral RNPs; thus, acidic HA activation is identified as an important determinant of influenza virus biology. In addition, its high degree of amino acid variation allows it to escape antibody recognition and constitutes a fundamental problem for vaccination strategies that are used to control infection. In this study we have addressed the possible contribution of HA S110L mutation to the high pathogenicity of F-IAV virus.

With this aim we characterized the HA properties that govern its biological activity and compared the virus expressing HA 110L (HA mut) with the corresponding wild type virus carrying HA 110S (CAL virus). No differences on glycosylation, the pH stability, or viral entry were observed (Figures 1D,E, 2, 3, respectively). However, recognition by monoclonal and polyclonal antibodies that attach to the HA protein from A/California/07/09 (Figures 1B,C) did show significant variations, suggesting a possible change in HA structure. In addition, the kinetics of the recombinant virus bearing the mutant HA protein was slightly delayed both, in single and multiple cycle growth curves in human epithelial cells (Figure 4). These data indicate that the mutation might partially debilitate the folding, processing or delivery of hemagglutinin to the plasma membrane, partially affecting its function. Furthermore, experiments performed in the mouse model, clearly showed that HA S110L mutation confers attenuation, since a high increase in the LD50 of the corresponding recombinant viruses was observed, even in the case of the virus expressing PA D529N, a particularly pathogenic virus (Figure 5B). The attenuation capacity of HA S110L mutation agrees with pathogenicity differences between PA mut and F-IAV virus. While the LD50 of PA mut is 3 × 10^3^, a 10^6^ viral dose is required to kill half of the mice infected with F-IAV virus (7); very likely HA S110L together with PB2 A221T mutation cooperated to diminish F-IAV pathogenicity. Alignment of all sequences available in the NCBI Influenza Resource database from April 2009 to March 2018 for HA of human H1N1 isolates shows that although HA at 110 position admits several changes including L, only 11 isolates from a total of 14,891 (<0.1%) sequences have an amino acid different than S, indicating that a serine at position 110 is required for efficient replication in humans (Table 2). According with the attenuated phenotype of HA mut, histological examination of the lungs of infected mice showed lower lung inflammation in the HA mut- than in CAL-infected mice (Figure 6C). The reduction in lung damage, seems to be the consequence of a low capacity of HA mut to localize to the bronchioli and lung parenchyma (Supplementary Figures 3J–L) and consequently it shows a general decrease on production of infective particles in the lungs (Figure 5C), compared with the wild type virus. Finally, the decreased replication capacity of HA mut in the lung also applied to its infection in leukocytes that was also reduced (Figure 7).

**Table 2:** Percentage of changes at position 110 of HA in A (H1N1) human viruses.

**A(H1N1) human viruses**	**HA 110**	**14891 sequences %**
2009–2018 circulating viruses	S	99.927
Amino acid change to L	L	0.047
Other amino acid changes	T/P	0.026
F virus	L	–

The *in vivo* data indicate that mutation HA S110L modifies HA protein, probably through some conformational change, reducing its capacity to reach the target cells in the context of the whole animal. All these data suggest that the attenuation mechanism might be related with viral ability to reach the lungs. Host-pathogen interactions promote co-evolution of defensive or invasive strategies, respectively. In the airways, inhaled respiratory viruses begin by facing the respiratory tract mucus, a selective biophysical barrier mainly composed by sialic-acid rich mucins [reviewed in (30)]. Mucins constitute decoys that are able to trap IAV viruses on account of the affinity to sialic acid displayed by hemagglutinin. This is an important defense mechanism counteracted by the viral neuraminidase able to cleave sialic acids to promote viral access to the epithelial layer (31, 32). As CAL and HA mut do not show variations on viral entry, the HA mut does not seem to affect sialic acid preference. However‘, there are many other host processes affecting the success of a virus to penetrate the airway epithelia that could participate in the attenuation of the HA mut. Increasing evidence suggest that interactions of the glycans of HA with membrane-bound and soluble lectins also are relevant for influenza virus infection. It has been shown that HA glycans bind to the surfactant protein D of the respiratory secretions (32–34) and to mannose-binding lectin present in serum (35). These compounds neutralize the virus using different mechanisms, such as steric burden of the receptor-binding site of HA, virus aggregation and inhibition and activation of complement-dependent pathways of the innate immune system; a potent yet unspecific mechanism that bridges the innate and adaptive immune system (20, 36). Additionally to the mucus layer, immunoglobulins such as IgA are present in mucosal epithelia, conferring innate protection from infection through neutralization of IAV virions at cell surface [(37) and reviewed in (38)]. Since HA is a glycoprotein exposed at the virion surface that interacts with many components of the host respiratory track; we can speculate that this plethora of defense mechanisms might be affected by subtle conformational changes on hemagglutinin inferred by the slight, but statistically significant difference observed in the neutralization assays (Figure 1B).

Taken together, we may speculate that mechanisms related with changes in viral tropism and/or recognition by host innate immunity sensors contribute to attenuation. In agreement, our data shows that HA-mut has reduced penetrance in the lungs (Figure 6), is better neutralized by the monoclonal and polyclonal antibodies tested (Figures 1B,C) and at day 2 post-infection recruits less neutrophils (Figure 7). These aspects, to which HA has been shown to contribute, are important determinants of the pathogenicity of a virus. HA has been well-documented in altering viral tropism. Receptor-binding specificity, differences in protease sensitivity of HA or in tissue specificity of the enzymes and HA glycosylation are important determinants of viral tropism in the respiratory tract and for spread of the virus (15, 39). HA-mut infects and spreads less in the lungs, which could indicate lower penetration in the respiratory tract, but could also be a downstream consequence of attenuation. Future experiments should determine whether CAL and HA-mut infect different population of cells. Interestingly, it was recently proposed that not only different influenza strains infect different cell types (including immune related cells), but also have opposite effects in the survival of these cells. In turn, these differences influence immune system recruitment and maturation (40). HA association with immunity has been shown to operate at different levels since the beginning of infection. As mentioned, virion entry in the airway epithelia faces many initial barriers, many of which are linked with HA, the major viral protein present in the viral envelope. Several interactions between the glycans of HA with host factors control innate immunity activation (41). Among these, interactions with lectins and with IgA control complement activation, viral clearance, and neutralization regulating the intensity of the immune response mounted to fight the microbial challenging (38). The better neutralization of the HA-mutant virus by monoclonal and polyclonal antibodies fits this box and could justify a reduction of viral titers, IFN activation and neutrophil recruitment. Infections of IgA deficient mice could help to clarify the role of this immunoglobulin. HA has also been linked to activation of immune response inside the cell. In this regards it was recently shown that ER stress pathway senses viral glycoproteins triggering innate immunity (42) and slight changes in the synthesis of viral RNA could impact in activation of interferon response (Figures 4E,F).

In sum, there are a variety of hurdles a virus needs to overcome while infecting a host. Differences, even if subtle, in the number of virions that infect the airway epithelia have a huge impact in the magnitude of the disease, controlling the penetration of the virus in the respiratory tract and ultimately dictating its final outcome.

## Author Contributions

AN designed the research studies, conducted databases research and wrote the manuscript. JV designed research studies, analyzed the data, and conducted experiments. NBS designed research studies, analyzed the data and conducted experiments. NZ and PL conducted experiments. MJA designed the research studies, analyzed the data and wrote the manuscript. AF designed the research studies, conducted the experiments, analyzed the data and wrote the manuscript.

### Conflict of Interest Statement

The authors declare that the research was conducted in the absence of any commercial or financial relationships that could be construed as a potential conflict of interest.

## Supplementary Material

Supplementary Figure 1 Location of amino acid changes in the 3D structure of HA. Residues with changes S88Y and K136N required simultaneously to inhibit binding of α-HA/Cal/2 to A/Cal/07/09 HA are indicated with orange color in the front subunit. Change T89K, which causes a fully resistant protein to neutralization by the antibody, is shown in blue. Figure modified from (12).

Supplementary Figure 2 Evaluation of pathogenesis of the recombinant influenza viruses in the mouse model. Mice (*n* = 5) were inoculated intranasally with indicated doses of the recombinant viruses or were mock-infected as control. Body weights were determined daily for 14 days (dpi) and mean body weight for each group infected mice is shown (*n* = 5/group).

Supplementary Figure 3 Immunological damage in lungs of CAL and HA mut-infected mice. Five Balb/c female mice/condition of 6–9 weeks of age were infected with 10^3^ pfu of CAL and HA-mut viruses or mock infected. At days 1, 2, and 3 post-infection lungs were collected, fixed with formalin, processed for histological analyses and stained with H&E. **(A–I)** show representaitive lungs at 1.25X amplification. Inlets are areas 10× amplified where specific damage (or its absence) is observed. ( Interstitial infiltrates;  Perivascular/peribronchioli infiltrates;  Bronchial exudates). Different inflammation and damage parameters **(J–M)** were graded on a scale 0–4 (0, absent; 1, very mild; 2, mild; 3, moderate; and 4, severe). Graphs are box-to-whiskers plots from min to max and line represents the median. Statistical analyses was done using two-way ANOVA and is indicated as ^*^*p* < 0.05; ^**^*p* < 0.01, ^***^*p* < 0.001 where significant differences were found. The experiment was performed twice.

Supplementary Figure 4 NP expression in lungs of CAL and HA mut-infected mice. Five Balb/c female mice/condition of 6–9 weeks of age were infected with 10^3^ pfu of CAL and HA-mut viruses or mock infected. At days 1,2, and 3 post-infection lungs were collected, fixed with formalin and processed for NP staining. **(A–I)** Show representaitive lungs at 1.25X amplification for indicated conditions. Inlets are areas 5–20× amplified where staining (or its absence) is observed. ( Perivascular/peribronchioli infected areas;  parenchyma areas infected). **(J–L)** NP expression in lungs was scored for the number and areas of infected bronchioli as follows: 1, 0–25% infected cells; 2, 25–50% infected cells; 3, 50–75% infected cells; 4, 75–100% infected cells. NP expression was also scored as present/absent infection foci on alveoli were scored 0 when absent or 1 if present. Graphs are box-to-whiskers plots from min to max and line represents the median. Statistical analyses was done using two-way ANOVA and is indicated as ^*^*p* < 0.05; ^**^*p* < 0.01, ^***^*p* < 0.001 where significant differences were found. The experiment was performed twice.
