# Supplementary figures and images for: Mutation S110L of H1N1 Influenza Virus Hemagglutinin: A Potent Determinant of Attenuation in the Mouse Model
Source: Front Immunol. 2019 Feb 6;10:132. doi: 10.3389/fimmu.2019.00132 (PMC6372558; doi:10.3389/fimmu.2019.00132)

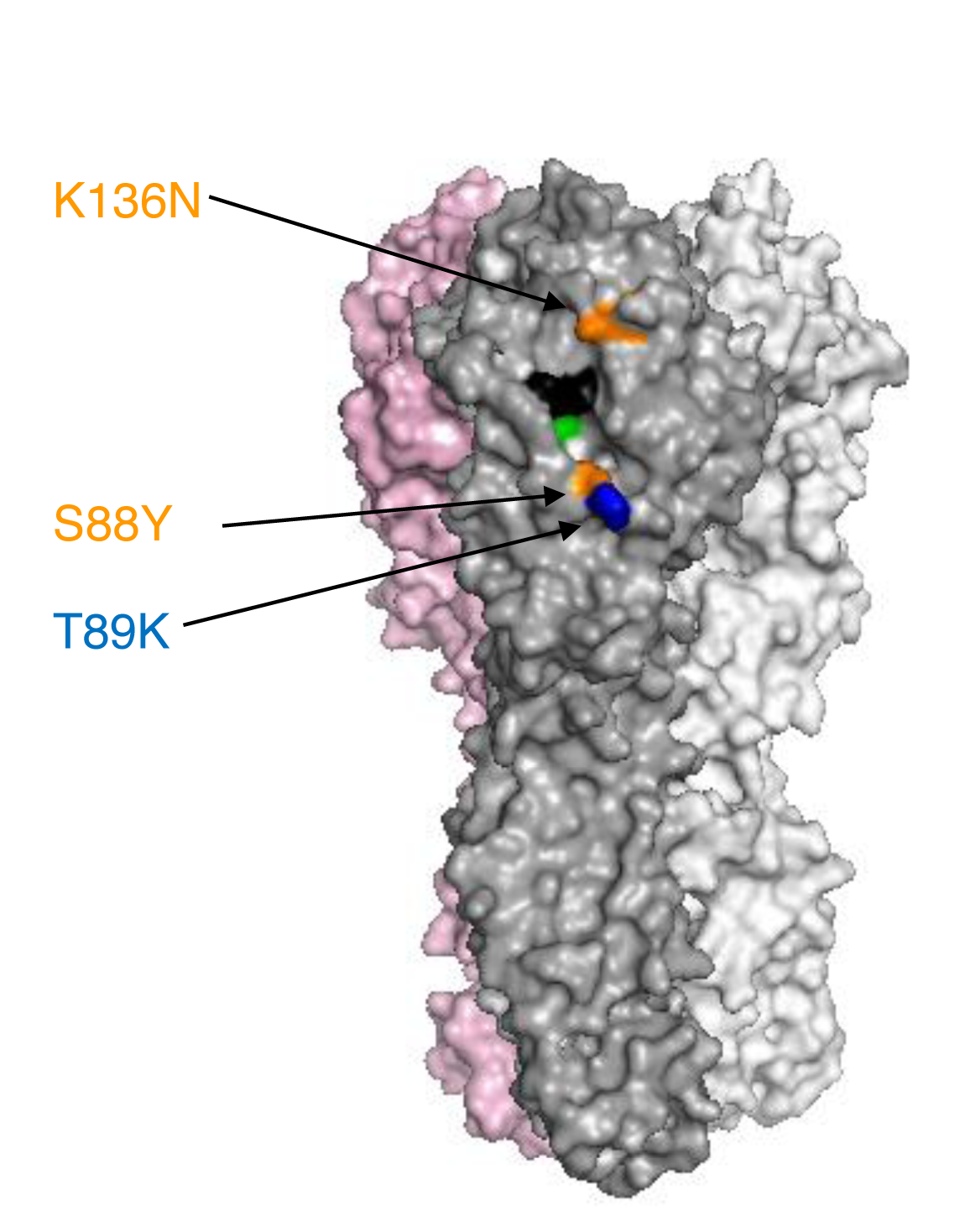

Supplement: Supplementary Figure 1 — Location of amino acid changes in the 3D structure of HA. Residues with changes S88Y and K136N required simultaneously to inhibit binding of α-HA/Cal/2 to A/Cal/07/09 HA are indicated with orange color in the front subunit. Change T89K, which causes a fully resistant protein to neutralization by the antibody, is shown in blue. Figure modified from (12). [file Image_1.TIF]

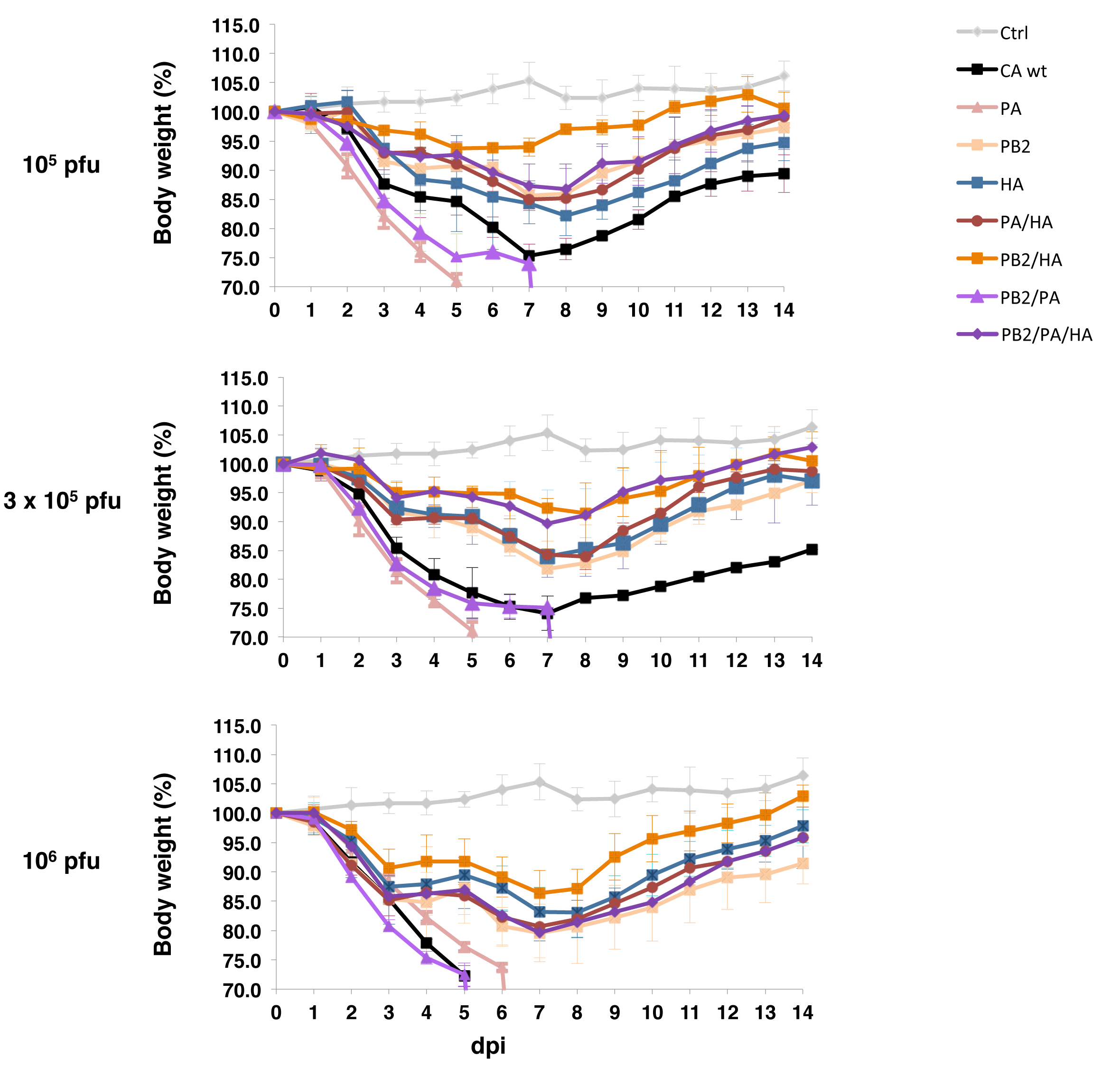

Supplement: Supplementary Figure 2 — Evaluation of pathogenesis of the recombinant influenza viruses in the mouse model. Mice (n = 5) were inoculated intranasally with indicated doses of the recombinant viruses or were mock-infected as control. Body weights were determined daily for 14 days (dpi) and mean body weight for each group infected mice is shown (n = 5/group). [file Image_2.TIF]

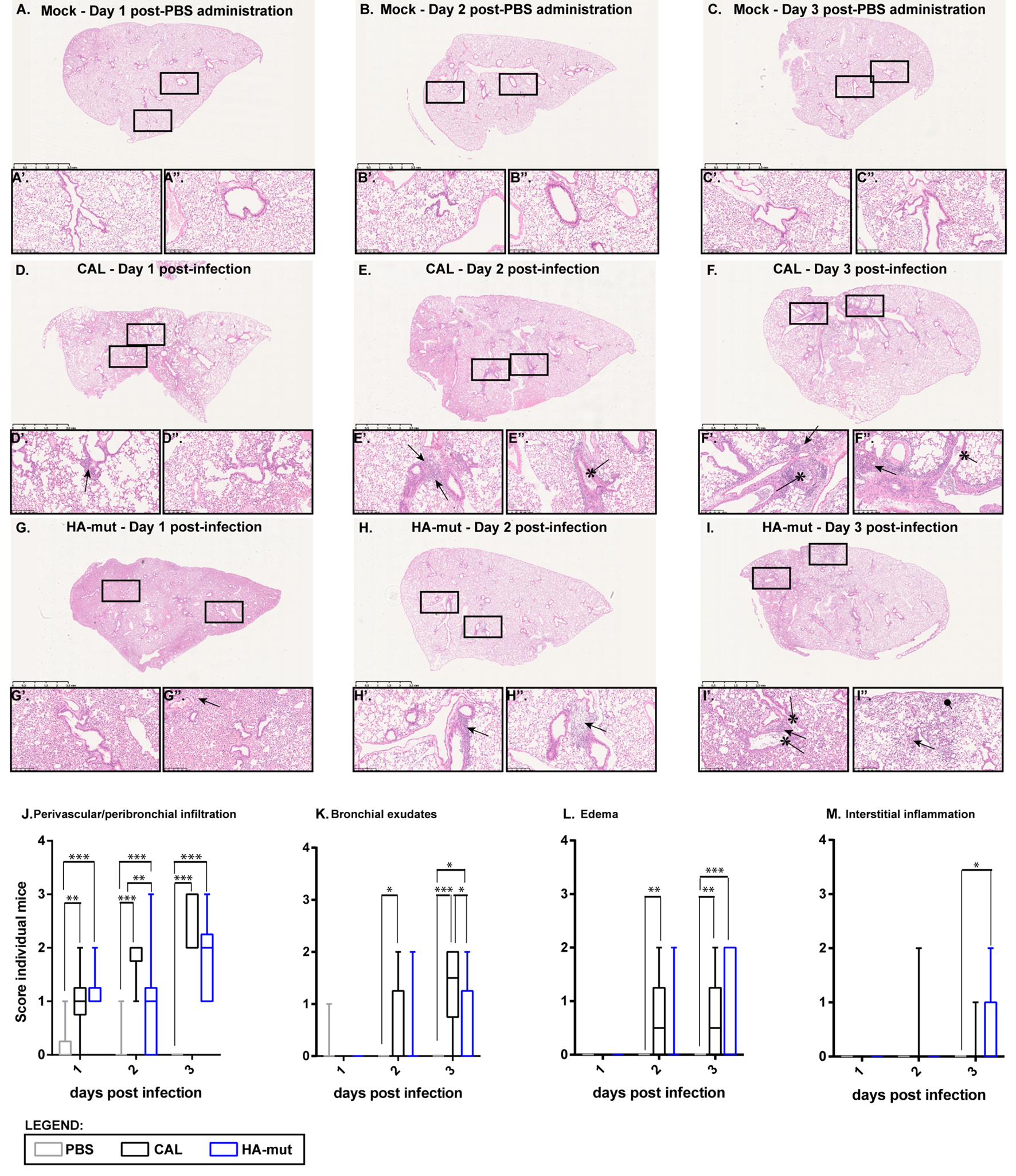

Supplement: Supplementary Figure 3 — Immunological damage in lungs of CAL and HA mut-infected mice. Five Balb/c female mice/condition of 6–9 weeks of age were infected with 103 pfu of CAL and HA-mut viruses or mock infected. At days 1, 2, and 3 post-infection lungs were collected, fixed with formalin, processed for histological analyses and stained with H&E. (A–I) show representaitive lungs at 1.25X amplification. Inlets are areas 10× amplified where specific damage (or its absence) is observed. ( Interstitial infiltrates; Perivascular/peribronchioli infiltrates; Bronchial exudates). Different inflammation and damage parameters (J–M) were graded on a scale 0–4 (0, absent; 1, very mild; 2, mild; 3, moderate; and 4, severe). Graphs are box-to-whiskers plots from min to max and line represents the median. Statistical analyses was done using two-way ANOVA and is indicated as *p < 0.05; **p < 0.01, ***p < 0.001 where significant differences were found. The experiment was performed twice. [file Image_3.TIF]

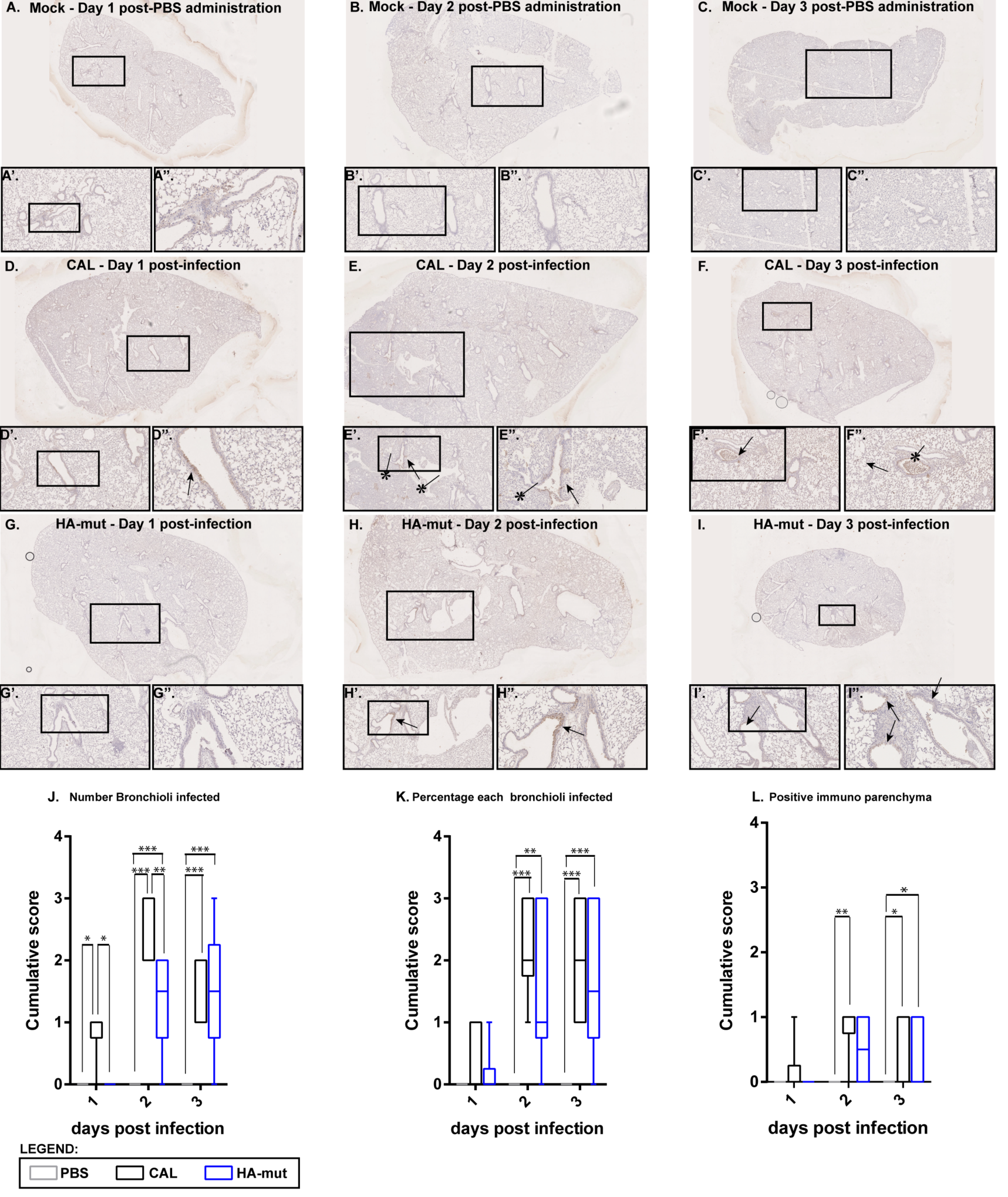

Supplement: Supplementary Figure 4 — NP expression in lungs of CAL and HA mut-infected mice. Five Balb/c female mice/condition of 6–9 weeks of age were infected with 103 pfu of CAL and HA-mut viruses or mock infected. At days 1,2, and 3 post-infection lungs were collected, fixed with formalin and processed for NP staining. (A–I) Show representaitive lungs at 1.25X amplification for indicated conditions. Inlets are areas 5–20× amplified where staining (or its absence) is observed. ( Perivascular/peribronchioli infected areas; parenchyma areas infected). (J–L) NP expression in lungs was scored for the number and areas of infected bronchioli as follows: 1, 0–25% infected cells; 2, 25–50% infected cells; 3, 50–75% infected cells; 4, 75–100% infected cells. NP expression was also scored as present/absent infection foci on alveoli were scored 0 when absent or 1 if present. Graphs are box-to-whiskers plots from min to max and line represents the median. Statistical analyses was done using two-way ANOVA and is indicated as *p < 0.05; **p < 0.01, ***p < 0.001 where significant differences were found. The experiment was performed twice. [file Image_4.TIF]
